# Mpox virus and transmission through sexual contact: Defining the research agenda

**DOI:** 10.1371/journal.pmed.1004163

**Published:** 2023-01-17

**Authors:** Nicola Low, Laura H. Bachmann, Dimie Ogoina, Robert McDonald, Aziz Mert Ipekci, Laura A. S. Quilter, Muge Cevik

**Affiliations:** 1 Institute of Social and Preventive Medicine, University of Bern, Bern, Switzerland; 2 Division of STD Prevention, Centers for Disease Control and Prevention, Atlanta, Georgia, United States of America; 3 Infectious Diseases Unit, Department of Internal Medicine, Niger Delta University/Niger Delta University Teaching Hospital, Bayelsa, Nigeria; 4 Graduate School for Health Sciences, University of Bern, Bern, Switzerland; 5 Division of Infection and Global Health Research, School of Medicine, University of St. Andrews, St. Andrews, Scotland, United Kingdom

## Abstract

In a Policy Forum piece, Dr. Nicola Low and colleagues define the research agenda for Mpox virus and transmission through sexual contact.

**Summary points**

- A multicountry outbreak of mpox (formerly monkeypox) in 2022 has affected gay, bisexual, and other men who have sex with men disproportionately, highlighting the need to understand the role of sexual contact in transmission.
- While mpox virus (mpoxv) is not a novel pathogen and has been circulating in sub-saharan Africa for many years, sexual transmission has not been well described.
- Epidemiological, clinical, and virological data indicate that transmission of mpoxv through sexual contact, both penetrative and nonpenetrative, is more effective than transmission through casual skin-to-skin contact.
- This article addresses many remaining questions about factors associated with the sexual route of mpoxv transmission and provides a framework within which research studies can be prioritised to guide policy and prevention needs.

A multicountry outbreak of mpox (formerly monkeypox) was first identified in Europe in May 2022 [1]. The number of new cases peaked in mid-August, followed by a rapid decline, which has continued in most affected countries. Countries with the largest numbers of cases have previously reported either no cases or sporadically imported cases from countries with endemic circulation. The outbreak has remained almost exclusively in social and sexual networks of gay, bisexual, and other men who have sex with men (GBMSM), raising many questions about the role of sexual contact in mpox virus (mpoxv) transmission. Many pathogens can be transmitted during sexual contact, mainly through genital fluids during sexual intercourse or through contact with skin or mucosal lesions. Mpox can be described as sexually transmissible, meaning that transmission can occur during a sexual encounter, with or without penetrative sexual intercourse. We do not address the question of whether mpox should be called “a sexually transmitted infection” (STI) or “a sexually transmitted disease” because those terms are inconsistently defined or applied in the literature. Understanding the role of sexual contact in mpoxv transmission remains a priority for both endemic and nonendemic countries to inform policy about research and prevention needs and to complement research recommendations about biomedical countermeasures [2]. We summarise here the epidemiological, clinical, and virological research on sexual transmission of mpoxv, discuss linked factors that might contribute to ongoing transmission, and propose priorities for research.

## Epidemiological findings supporting mpoxv transmission through sexual contact

The first reports of mpox in the 2022 outbreak were men identifying as GBMSM who presented with mucosal genital lesions [3–6] and reported sexual contact as a route of potential transmission. The rapid evolution is consistent with transmission in closely connected social and sexual networks of GBMSM, within which high rates of sexual partner change are reported [7]. Travel to large GBMSM events in Spain and Portugal likely amplified onward transmission in many countries [7,8]. A mathematical modelling study, using data about sexual partner numbers in Great Britain, found that even one event of sexually associated mpoxv in a GBMSM population resulted in a high probability of a large outbreak, but that transmission within non-MSM networks would be unlikely to be sustained [9]. The small number of cases among women and children in all countries supports the low risk of sustained transmission outside GBMSM networks.

Mpoxv is not a new pathogen in humans and nonsexual transmission routes are well described [10]. The potential for human-to-human transmission of orthopoxviruses through sexual contact is established in reports of vaccine-strain vaccinia virus transmission [11]. In addition, during an outbreak in Nigeria that started in 2017, sexual transmission was suggested [12]. Unlike previous outbreaks, most cases were males (84/122, 69%) living in urban areas and few reported direct contact with animals. Most men presented with genital lesions (44/65, 68%) and several had coinfections with HIV or syphilis [13]. Some men reported sexual behaviours that would facilitate sexual transmission, but none reported male sexual partners [12]. The illegality of male same sex sexual contact in many countries in sub-Saharan Africa is a barrier to disclosure during medical consultations and to research about sexual orientation, making it challenging to establish sexual transmission of mpoxv.

## Clinical findings supporting mpoxv transmission through sexual contact

The clinical presentation of mpoxv indicated a role for direct inoculation during sexual contact very early on [3–6,8,14]. Almost all cases identified as GBMSM and had mucosal genital lesions (Table 1). Some presented with solitary genital lesions, which were misdiagnosed as STIs such as herpes simplex, disseminated gonorrhoea, or syphilis [4,8]. In one couple, the location of lesions was consistent with their insertive and receptive sexual roles [14]. From 14% (75/528) to 36% (71/197) presented with rectal pain or proctitis, prominent features of the illness, and those who reported receptive anal sex were more likely to have proctitis (41/108, 38% versus 4/58, 7%) [6]. Concurrent bacterial STI were diagnosed in 17% (30/181) to 76% (140/185), and 24% (13/54) to 44% (225/508) were living with HIV (Table 1) [3–6,15–17].

**Table 1:** Clinical and behavioural findings from mpox case series with more than 50 cases published in July 2022, by date of publication.

First author^a^	Girometti N [5]	Thornhill JP [3]	Patel A [4]	Tarín-Vicente EJ [6]	Catala A [15]	Inigo Martinez J [17]
Date	14–25 May 2022	27 April–24 June 2022	13 May −1 July 2022	11 May −29 June 2022	28 May −14 July 2022	17 May −22 June 2022
Number of cases	54	528	197	181	185	508
Locations	1 sexual health clinic, London	43 clinical sites, 16 countries	1 sexual health clinic, London	3 sexual health clinics, Madrid, Barcelona	Medical facilities, Spain	Sexual health clinics and hospitals, Madrid
Male, n (%)	54 (100)	527 (100)	197 (100)	175 (97)	185 (100)	503 (99)
GBMSM, n (%)	54 (100)	519 (98)	196 (100)	166 (97)	184 (99)	397 (93)
Age in years, median (IQR or range) or mean (SD)	41 (IQR 34–45)	38 (range 18–68)	38 (IQR 32–42)	37 (IQR 31–42)	38.7 (SD 8.2)	35 (range 18–67)
Travel history, n (%), timing	25 (46), last 2 months	147 (28), 4 weeks before diagnosis	54 (27), 4 weeks before symptoms	26 (14), ‘recent’ outside Spain	51 (28), 3 weeks outside home town	38 (8), last 3 weeks
Sex partners last 3 months, n (%) or median (IQR)	29 (56%) >5 partners	5 (3–15)	Not reported	6·5 (3–16)	8 (4–17)	Not reported
Concurrent STI,^b^ n (%)	13/51 (25)	109/377 (29)	56/178 (32)	30/181 (17)	140/185 (76)	Not reported
Living with HIV, n (%)	13 (24)	218 (41)	70 (36)	73 (40)	78 (42)	225 (44)
First symptom						
Systemic	44/54 (81)	17/30 (57)	102/166 (62)	87 (48)	185 (100)	Not reported
Rash	10 (19)	13/30 (43)	64/166 (39)	Not reported	118 (64)	Not reported
Single anogenital lesion	Not reported	54 (10)	22 (11)	Not reported	21 (11)	Not reported
Lesion numbers, n (%) or median (IQR)	Not reported	≤20, 450 (85)	5 (3–11)	≤20, 160 (88)	≤ 25, 173 (93)	Not reported
Genital	51 (94)	383 (73)	111 (56)	141 (78)	98 (53)	359 (72)
Proctitis/anorectal pain	Not reported	75 (14)	71 (36)	45 (25)	40 (22)	81 (16)
Hospitalised, n (%)	5 (9)	70 (13)	20 (10)	2 (1)	4 (2)	19 (4)
Deaths, n	0	0	0	0	0	0

GBMSM, gay, bisexual and other men who have sex with men; IQR, interquartile range; SD, standard deviation; STI, sexually transmitted infection.

^a^There may be overlap between the cases reported in these studies, since some authors appear in more than one publication.

^b^Girometti and colleagues, gonorrhoea or chlamydia; Thornhill and colleagues, gonorrhoea, chlamydia, or syphilis; Patel and colleagues, gonorrhoea, chlamydia, herpes simplex, syphilis; Tarín-Vicente, gonorrhoea, chlamydia, herpes simplex, *Mycoplasma genitalium*, syphilis; Catala and colleagues, not specified.

## Virological findings supporting transmission through sexual contact

Mpoxv DNA was detected by polymerase chain reaction (PCR) in the semen of 29 of 32 tested cases at presentation in an early international study, suggesting the potential for transmission via genital secretions [3]. In a Spanish cohort, different bodily specimens were analysed for up to 57 days since symptom onset in 74 patients [18]. Mpoxv detection by PCR was longer, and viral loads higher, in samples from lesions than from pharyngeal swabs or semen, highlighting the importance of intimate skin-to-skin contact. In 78 samples tested by viral culture, mpoxv was less commonly detected in semen and pharyngeal samples than from lesions, suggesting more frequent transmission via intimate contact with infectious lesions than respiratory droplets or semen [18].

Phylogenetic analyses suggest a nonhuman animal to human spillover and a new mpoxv clade (IIb), which was likely transmitted undetected before the recognised outbreak in May 2022 [19]. Genomic analyses of enzyme (APOBEC3) mutations are being conducted to investigate the possibility of viral evolution with enhanced human-to-human transmissibility [20], which appears to have become more efficient from around 2017.

## Research priorities for investigating the role of sexual contact in mpoxv transmission

**Fig 1:**
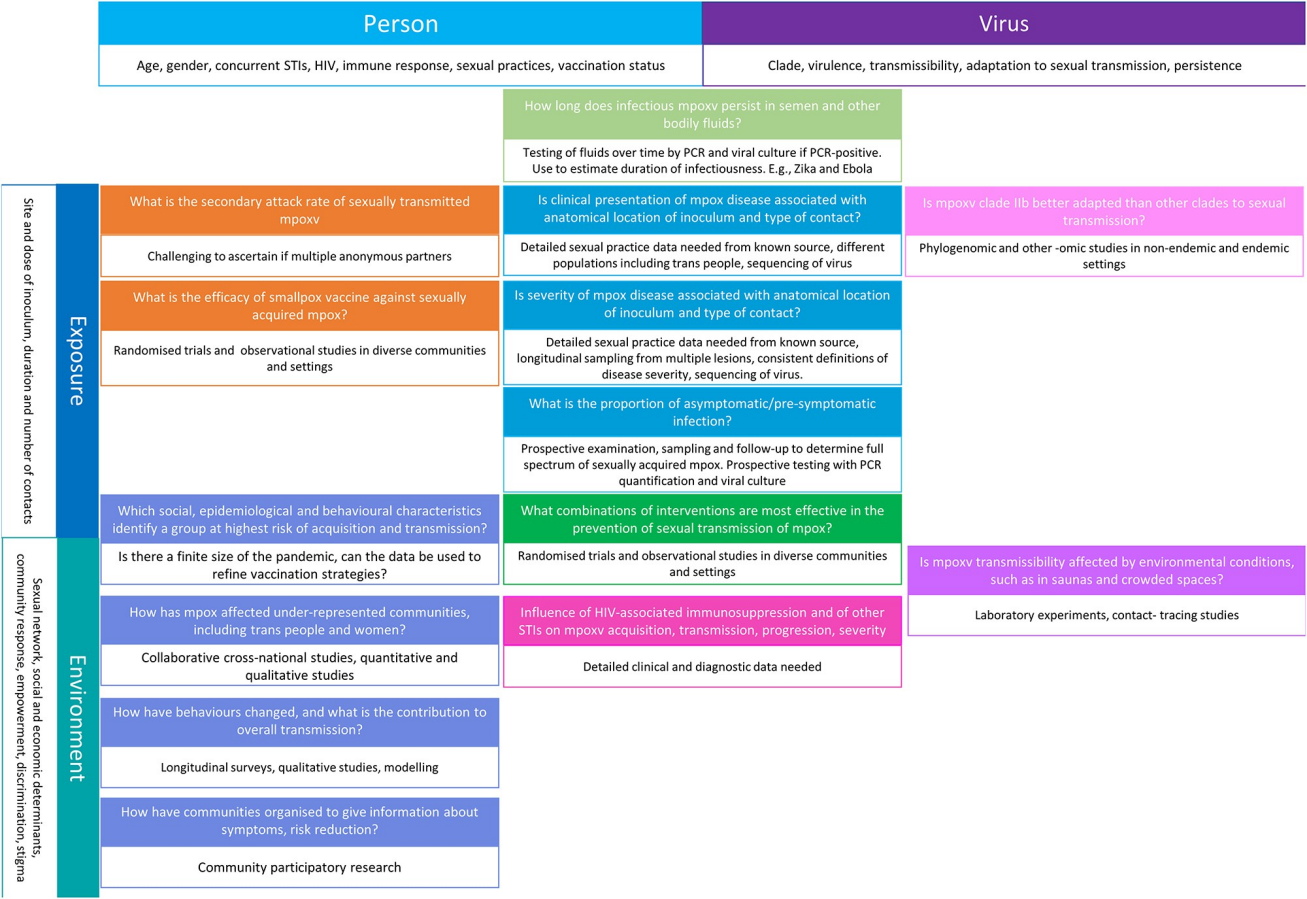
Research questions and factors that could influence the transmission of mpoxv through sexual contact, alone or in combination. The framework includes 4 domains of factors that could affect transmission at the top (the person and the virus) and left-hand side (the exposure and the environment) of the figure, with specific factors listed in the box below the domain heading. Research questions are inside the grid. They may affect or be affected by multiple factors, shown by their position in the framework. S1 Table lists the research questions. mpoxv, mpox virus; PCR, polymerase chain reaction; STI, sexually transmitted infection.

Disproportionately high incidence rates among GBMSM and research findings to date show the need for additional studies to understand the role of sexual contact in mpoxv transmission and guide interventions that can sustain the decline in sexually transmitted mpoxv. Fig 1 shows a framework of factors in 4 domains, which may influence mpoxv transmission: the person, exposure, virus, and environment. Fig 1 also details research questions and where they fit into framework domains. Selected questions are discussed in more detail and summarised in S1 Table. Research studies will require multidisciplinary expertise in clinical science, epidemiology, phylogenomics, virology, mathematical modelling, and social science. Multicountry collaborative studies, involving existing research cohorts, using harmonised protocols, will help to provide rapid, actionable, and sustainable results.

### Is clinical presentation of mpoxv disease associated with anatomical location of inoculum and type of contact?

The location of the inoculum at different types of cutaneous surfaces, e.g., keratinised squamous or columnar mucosa and local immune responses, could influence the spectrum of disease. In a Spanish cohort, proctitis was more common when reporting anal receptive intercourse and tonsillitis with oral receptive intercourse [6]. Elucidation of associations between sexual contact type and clinical presentation is challenging, especially with multiple exposures in a short period or at different types of venues within a close network. Smaller studies with detailed data about types and intensity of exposures [14] might be more appropriate to answer this question than larger studies with less detailed data. These studies could also generate estimates of incubation period for different types of sexual practice.

### Is severity of mpoxv disease associated with anatomical location and dose of inoculum?

The severity of mpoxv disease in the current outbreak might also be influenced by the anatomical location of exposure and dose of inoculum. An association between route of acquisition of mpoxv and severity of disease was reported in an outbreak in the United States of America [21]. While there were no cases of human-to-human transmission, individuals with a “complex” exposure (an animal bite or scratch) were more likely to have severe clinical illness (8/17) than those with “noninvasive” exposures (mucosal or respiratory exposures, 5/30). Condomless anal intercourse or other practices that cause abrasions or microabrasions during sex could be analogous to a “complex” exposure.

Longitudinal studies, containing detailed data about the likely anatomical location of inoculation and type of contact and about the types and timing of symptom onset, with follow-up throughout the course of infection to monitor the evolution and resolution of disease, are needed. Longitudinal sampling from multiple lesions, with virological and phylogenomic analyses, will allow studies of virus evolution related to adaptation of mpoxv to human transmission [22].

### What is the proportion of asymptomatic or presymptomatic infection in the context of sexual transmission?

Asymptomatic or presymptomatic mpoxv infection has implications for prevention of transmission. Infections with few or atypical symptoms may facilitate transmission, especially if lesions are not noticed during sexual contact. Mpoxv was detected by PCR in 3/224 1.3% (95% confidence interval (CI) 0.3% to 3.9%) anorectal samples, from GBMSM with no reported symptoms [23]. In another study, mpoxv PCR was positive in 13 of 200 anorectal samples from GBMSM reporting no symptoms at the time of testing. Two subsequently developed symptoms, suggesting 11/200 (5.5%, 95% CI 2.8% to 9.6%) had persistently asymptomatic infection [24]. These studies were retrospective with no physical examination at the time, so small or atypical lesions could have been missed. Mathematical modelling studies suggest that asymptomatic, presymptomatic, or undiagnosed mpoxv have likely contributed substantially to epidemics in Belgium and the United Kingdom [25,26].

Studies to ascertain persistently asymptomatic infection need to be prospective, with enrolment that is not influenced by the presence or absence of symptoms. Studies of asymptomatic SARS-CoV-2 have shown the need to reduce risks of selection and information biases [27]. Seroprevalence studies conducted early in an epidemic among people born after smallpox vaccination ceased in the 1970s to 1980s, with careful symptom histories, can also help to determine the prevalence of asymptomatic infection.

### Persistence of mpoxv in semen and other bodily fluids

The clinical significance of mpoxv in semen is still unclear due to difficulty in distinguishing transmission through infectious virus in genital fluids or from mucosal lesions [3]. Cohort study findings suggest that the risk of transmission through semen is less than through lesions [18]. No studies have investigated the testis or prostate as sanctuary sites yet. Researchers have published study protocols for cohort studies of the persistence of virus in semen, urine, vaginal fluid, and other bodily fluids in Zika [28] and Ebola [29] infections, and these can be adapted to study mpoxv. Such studies can inform guidance about the potential value and duration of condom use after recovery from infection.

### HIV infection and other STIs

Sizeable proportions of patients with mpoxv were reported to have concurrent STIs and/or were living with HIV infection [3–6]. HIV infection was not associated with higher rates of severe mpox in patients with well-controlled HIV infection. In Nigeria and the USA, people living with HIV and profound immunosuppression have been disproportionately represented among those with severe and complex illness, who were hospitalised, and, in Nigeria, among those who died [13,16]. The influence of HIV infection, other STIs and immunosuppression on mpoxv acquisition, transmission, and clinical progression needs further investigation.

### Transmission dynamics of mpoxv

Clinical and epidemiological data can be used to improve estimates of the number of GBMSM at highest risk of acquiring and transmitting mpoxv and the transmissibility of mpoxv. Together with data about asymptomatic infection, incubation period, and secondary attack rate, mathematical modelling studies will be better able to determine the contributions of infection-induced immunity and behavioural change to the epidemic dynamics [9], and the impact of vaccination strategies or other mitigation and countermeasures.

### Community response, sexual behaviour change, discrimination, and stigmatisation

GBMSM as members and leaders of various professions, as activists, and as individuals have taken the initiative to inform themselves and their communities about mpox [30]. Community-led campaigns have provided information about risks of infection acquisition and transmission, care-seeking, and vaccination. Quantitative and qualitative studies will need to investigate a range of questions, including how sexual behaviours and practices have changed and the experience and impact of discrimination and stigmatisation on sexual behaviours.

## Conclusions

The world’s largest multicountry outbreak of mpoxv was sustained in social and sexual networks of GBMSM. Experience from previous pandemics, including HIV, Zika, Ebola, and COVID-19, should be applied to understand transmission and prevention of mpoxv in a wide range of communities, including transgender people and other underrepresented populations. Understanding the factors that have contributed to the origins and ongoing pattern of transmission through sexual contact is essential for the equitable implementation of existing prevention and control measures and development of new interventions, for current and future outbreaks.

## Supporting information

S1 Table Research priorities for questions about mpox in the context of sexual contact, according to domains of factors associated with mpox virus transmission. (DOCX)

## Abbreviations

CI: confidence interval
GBMSM: gay, bisexual, and other men who have sex with men
mpoxv: mpox virus
PCR: polymerase chain reaction
STI: sexually transmitted infection
