# Supplementary material for: Mpox virus and transmission through sexual contact: Defining the research agenda
Source: PLoS Med. 2023 Jan 17;20(1):e1004163. doi: 10.1371/journal.pmed.1004163 (PMC9888714; doi:10.1371/journal.pmed.1004163)
Supplement: S1 Table — (DOCX) [file pmed.1004163.s001.docx]

**S1 Table.** Research priorities for questions about mpox in the context of sexual contact, according to domains of factors associated with mpox virus transmission

| **Main domain(s) of factors associated with mpoxv sexual transmission**^a^ | **Research question**^b^ | **Comments and notes** |
| --- | --- | --- |
| Person, virus | How long does infectious mpoxv persist in lesions, semen and other bodily fluids? | Testing of fluids over time by PCR and viral culture if PCR-positive. Use to estimate duration of infectiousness. Examples from Zika and Ebola virus research are available |
| Person, virus, exposure | Is clinical presentation of mpox disease associated with anatomical location of inoculum and type of contact? | Detailed sexual practice data needed from known source. Sequencing of virus. |
| Person, virus, exposure | Is severity of mpox disease associated with anatomical location and dose of inoculum? | Longitudinal sampling from multiple lesions, consistent definitions of disease severity needed. Sequencing of virus. |
| Person, virus, exposure | How common are paucisymptomatic or ‘atypical’ presentations of mpox? | Prospective examination, sampling and follow-up to determine full spectrum of sexually acquired mpox. Compare endemic and non-endemic settings |
| Person, virus, exposure | What is the proportion of asymptomatic or presymptomatic sexual infection? | History and examination for symptoms and signs, Prospective testing with PCR quantification and viral culture to determine infectious potential, and follow-up that covers the full Incubation period. |
| Person, virus, exposure | What combinations of interventions are most effective in the prevention of sexual transmission of mpox? | Randomised trials and observational studies in diverse communities and settings; mathematical modelling |
| Person, virus, environment | What is the influence of HIV-associated immunosuppression and of other STIs on mpoxv acquisition, transmission, progression and severity? | Detailed information on co-infections, markers of immunity and viral load, as well as clinical and behavioural data. Compare diverse communities and settings. |
| Virus, exposure | Is mpoxv clade Iib better adapted to sexual transmission than other clades? | Phylogenomic and other -omic studies in non-endemic and endemic settings |
| Person, exposure | What is the secondary attack rate of sexually transmitted mpoxv | Challenging to ascertain if multiple anonymous partners |
| Person, exposure | What is the efficacy of smallpox vaccine against sexually acquired mpox? | Randomised trials optimal, but observational comparative effectiveness studies more likely |
| Person, exposure | Does smallpox vaccination prevent re-infection through sexual contact with mpox? | Vaccine strategy, e.g. single dose, fractional dosing might modify effect |
| Person, environment | Are there social, epidemiological and behavioural characteristics that identify a core group at highest risk of acquisition and transmission? | Is there a finite size of the pandemic, can the data be used to refine vaccination strategies? |
| Person, environment | How has mpox affected under-represented communities, including trans people and women? | Collaborative cross-national studies, quantitative and qualitative studies |
| Person, environment | How have sexual behaviours changed during the mpox pandemic? | Longitudinal surveys in existing cohorts, including people taking pre-exposure prophylaxis and people living with HIV. Qualitative and modelling studies too |
| Person, environment | How have communities organised to give information about symptoms, risk reduction? | Community participatory research in diverse communities and settings |
| Person, environment | What are people’s experiences of discrimination by sexual orientation, ethnic group? | Qualitative and quantitative studies, community-led, intersectionality |
| Virus, environment | Is virus transmissibility affected by environmental conditions, such as in saunas and crowded spaces? | Data required from laboratory experiments, contact tracing studies |

1. Colour of text corresponds to Fig 1 colours assigned to 4 domains of factors associated with mpoxv transmission in the context of sexual contact;
2. Colour of boxes corresponds to Fig 1 colours assigned to groups of research questions.
